# *Ascophyllum nodosum* Extract and Glycine Betaine Preharvest Application in Grapevine: Enhancement of Berry Quality, Phytochemical Content and Antioxidant Properties

**DOI:** 10.3390/antiox12101835

**Published:** 2023-10-07

**Authors:** Eliana Monteiro, Miguel Baltazar, Sandra Pereira, Sofia Correia, Helena Ferreira, Fernando Alves, Isabel Cortez, Isaura Castro, Berta Gonçalves

**Affiliations:** 1 Centre for the Research and Technology of Agro-Environmental and Biological Sciences (CITAB), University of Trás-os-Montes e Alto Douro (UTAD), 5000-801 Vila Real, Portugal; elianaribeiromonteiro@hotmail.com (E.M.); migueladbaltazar@gmail.com (M.B.); sirp@utad.pt (S.P.); sofiacorreia@utad.pt (S.C.); helenaf@utad.pt (H.F.); icortez@utad.pt (I.C.); icastro@utad.pt (I.C.); 2 Institute for Innovation, Capacity Building and Sustainability of Agri-food Production (Inov4Agro), University of Trás-os-Montes e Alto Douro (UTAD), 5000-801 Vila Real, Portugal; 3 Symington Family Estates, Vinhos SA, Travessa Barão de Forrester 86, 4431-901 Vila Nova de Gaia, Portugal; fernando.alves@symington.com; 4 Department of Agronomy, University of Trás-os-Montes e Alto Douro (UTAD), 5000-801 Vila Real, Portugal; 5 Department of Genetics and Biotechnology, University of Trás-os-Montes e Alto Douro (UTAD), 5000-801 Vila Real, Portugal; 6 Department of Biology and Environment, University of Trás-os-Montes e Alto Douro (UTAD), 5000-801 Vila Real, Portugal

**Keywords:** antioxidant capacity, bioactive compounds, biostimulants, climate change, grapevine quality, sustainable viticulture, *Vitis vinifera* L.

## Abstract

The Douro Demarcated Region (DDR) has peculiar edaphoclimatic characteristics that provide a suitable terroir for premium wine production. As climate change effects continue to emerge, ensuring productivity and quality becomes increasingly important for viticulturists, as those directly determine their profits. Cultural approaches, such as the use of biostimulants, are actively being developed to mitigate abiotic stress. The main objective of this work was to assess the effect of foliar sprays of a seaweed (*Ascophyllum nodosum*)-based extract (ANE) and glycine betaine (GB) on grape berry quality, bioactive compounds, and antioxidant activity. A trial was installed in a commercial vineyard (cv. ‘Touriga Franca’) in the *Douro Superior* (Upper Douro) sub-region of the Douro Demarcated Region. In 2020 and 2021, three foliar sprayings were performed during the growing season, namely at pea size, bunch closure, and veraison. There was a positive effect of both biostimulants (ANE and GB) on the physiological and biochemical performance of cv. ‘Touriga Franca’ exposed to summer stress. In general, the GB 0.2% spraying was the most promising treatment for this grape cultivar, as it increased berry quality, the concentration of bioactive compounds (total phenolics, flavonoids, and *ortho*-diphenols), and the antioxidant activity. These results revealed the efficacy of biostimulant sprayings as a sustainable viticultural practice, improving berry quality under summer stress conditions.

## 1. Introduction

Climatic conditions are major factors influencing the quality of grapes and wine. According to OIV [1], between 2020 and 2021, wine production in the EU declined by around 8%, which was attributed to the extreme differences in weather conditions throughout the years. Despite this, the European countries Italy, France, and Spain were the top three wine-producing countries in 2021, accounting for 47% of the world’s wine production [1]. However, this production is expected to be affected by the prominent negative impacts of climate change on grapevine physiology, growth, yield, and berry quality [2,3,4]. In order to prevent this, climate change mitigation strategies, such as the use of biostimulants, are increasingly needed [5,6,7,8,9,10,11,12,13,14,15]. Biostimulants, including *Ascophyllum nodosum* extracts and glycine betaine, are widely used in grapevines and in many other crops, such as sweet cherry, strawberry, hazelnuts, cowpea, alfalfa, and sweet potato [5,6,7,8,9,10,11,12,13,14,15]. Brown seaweed extracts are amongst the most used biostimulants in agriculture, with *A. nodosum* L. extracts being the most studied. These seaweed extracts have been described as being able to improve berry quality by regulating molecular, physiological, and biochemical processes [8,11,12,16,17]. Glycine betaine has also been reported as one of the most attractive biostimulants for plant stress protection, as it is naturally synthesized, non-toxic, and inexpensive [18]. Moreover, according to the literature, this compound can act as an osmoprotectant, maintaining cellular osmolarity, protecting photosynthetic machinery (photosystem II) and thylakoid membranes, alleviating cellular oxidative damage, and stabilizing protein structures [18,19]. Moreover, the new European Union (EU) Fertilizing Products Regulation 2019/1009 (EU, 2019) recognizes plant biostimulants as a distinct category of agricultural inputs, opening new opportunities for the use of these products in agriculture. The foliar application of biostimulants is a sustainable solution, given their natural origin and their potential to replace conventional methods in agriculture [17]. Biostimulants improve plant growth and nutrient absorption and are also an alternative to soil fertilization, avoiding some of the negative effects on the environment resulting from the leaching of nutrients into groundwater [5].

As climate change threatens worldwide wine production, there is a need to understand how mitigation strategies, such as the use of biostimulants, can be effective under field conditions. Therefore, the aim of this study was to evaluate the effect of a seaweed-based biostimulant (*A. nodosum*) and glycine betaine on berry quality, bioactive compounds, and antioxidant activity in *Vitis vinifera* L. cv. ‘Touriga Franca’. ‘Touriga Franca’ is the most cultivated grape variety in the Douro Demarcated Region, the oldest regulated wine denomination in the world characterized by a peculiar terroir with cold winters and very dry and hot summers, which is severely affected by climate change.

## 2. Materials and Methods

### 2.1. Plant Material and Sampling

**Figure 1:**
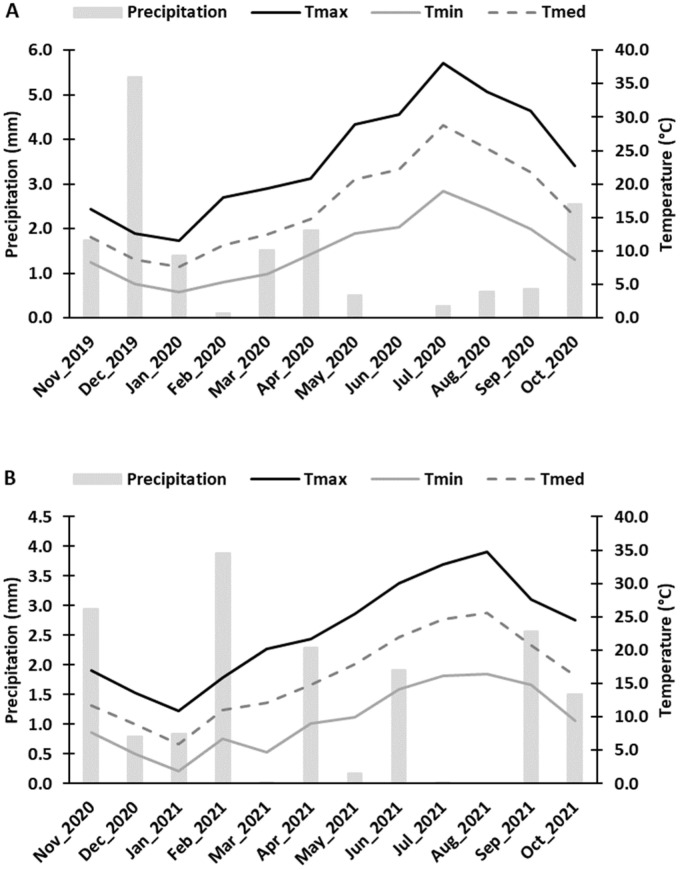
Monthly mean climatic conditions occurred during 2020 (**A**) and 2021 (**B**) growing seasons in the vineyard Quinta do Ataíde in the *Douro Superior* sub-region. Precipitation (mm); Maximum temperature—Tmax (°C); Minimum temperature—Tmin (°C) and Mean temperature—Tmed (°C).

The trial was installed in an organic commercial vineyard planted in 2013, in the *Douro Superior* (Upper Douro) sub-region of the Douro Demarcated Region, Vila Flor, Portugal (41°15′03.3″ N 7°06′38.7″ W, 160 m above sea level). Samples were obtained from the black-skinned *Vitis vinifera* cv. ‘Touriga Franca’ grafted on 196-17 C rootstock, in two growing seasons: 2020 and 2021. ‘Touriga Franca’ is the most cultivated variety in this region (27.3% of the total vineyard area), and the second (8%) is in Portugal [20]. Row and vine spacing was 2.10 m and 0.9 m, respectively, and vines were trained to unilateral Royat Cordon, with vertical shoot positioning (VSP) in an east–southeast to west–northwest orientation. The vineyard was drip-irrigated weekly, with a 30% replacement of the ETc (crop evapotranspiration), from bunch closure until two weeks before harvest. The climatic characteristics of this parcel consist of cold winters, with several days having minimum temperatures below 0 °C, and dry and hot summers. Monthly temperature and precipitation values were recorded via a weather station located near the experimental site and are shown in Figure 1.

In 2020 and 2021, three foliar sprayings were performed during the growing seasons, namely at pea size (BBCH 75), bunch closure (BBCH 77), and veraison (BBCH 81) [21]. Foliar applications were carried out during the morning, covering the whole canopy. The treatments tested were *A*. *nodosum* seaweed-based extract (SPRINTEX NEW^®^ L, Biolchim (Bologna, Italy), containing a high concentration of *naphthaleneacetic acid*, *amino acids*, and extract of *A. nodosum*) (ANE) at two different concentrations (ANE 0.05% and ANE 0.1%), glycine betaine (Greenstim^®^, Massó Agro Department (Barcelona, Spain), containing (*w*/*w*) 12% of total N, 11.5% organic N, 56% organic C and a relation C/N of 4.9, has a concentrate of glycine betaine extracted from sugar beet) (GB) at two different concentrations (GB 0.1% and GB 0.2%), and control (C, water) (5 treatments × 10 plants × 3 replicates). For all the solutions used in the foliar applications, a wetting agent (0.1%) was added. SPRINTEX NEW^®^ L and Greenstim^®^ were commercialized according to the national legislation decree-law 103/2015 of 15 June. Currently, only Greenstim^®^ is part of the list of non-harmonized fertilizing materials authorized for organic viticulture with registration valid until 2028 as requested by EU and national regulations (EU 2019/109 of 5 June and Ordinance 185/2022 of 21 July), respectively. At veraison (BBCH 81) and harvest (BBCH 89) [21], around 90 berries per treatment (divided into three replicates) were randomly sampled from the middle section of the bunches for quality analysis. Additionally, for the bioactive compounds and antioxidant activity determinations, three replicates of berries per treatment were sampled and immediately frozen in liquid nitrogen until conservation at −80 °C and then lyophilized and converted to a fine-dried powder (ground with liquid nitrogen) before laboratory analysis.

### 2.2. Quality Assessment of Fruits

Biometric parameters (berry weight and dimensions), color, total soluble solids, pH, titratable acidity, and maturity index were determined in 90 fruits per treatment condition, divided into three replicates, and sampled at veraison and harvest stages. Fruit weight (g) was determined using an electronic balance and using a digital caliper (0.01 mm sensitivity). The height (mm), width (mm), and thickness (mm) were measured. The external fruit color was assessed with a colorimeter (CR-300, Minolta, Osaka, Japan), previously calibrated using a standard white plate. With the colorimetric coordinates, where L* indicates lightness, a* indicates red (+ a) to green (− a) colors, and b* indicates yellow (+ b) to blue (− b) colors, chroma (C*) value was calculated using the formula C* = (a*^2^ + b*^2^)^1/2^. Measurements were taken from two opposite sides of each fruit. After these analyses, the 30 berries were divided into three groups of ten fruits, which were then macerated with a mortar and pestle to obtain juice. The total soluble solids (TSS in °Brix) of each berry juice were determined with a portable refractometer (PAL-1, ATAGO, Tokyo, Japan), and pH was measured using a portable pH meter (Hanna instrument, Woonsocket, RI, USA). Titratable acidity (TA) (gL^−1^ tartaric acid) was determined on 10 mL of juice diluted in 10 mL distilled water using a manual glass burette with 0.1 M NaOH to an endpoint of pH 8.1. The maturity index (MI) was calculated using the formula: MI = TSSpH^2^ [22].

### 2.3. Determination of Bioactive Compounds

For sample extraction, 950 µL of 70% (*v*/*v*) methanol were added to 40 mg of dry material of each berry sample and mixed thoroughly in a vortex. After that, the mixture was submitted to 70 °C for 30 min and finally centrifuged at 13,000 rpm at 1 °C for 15 min. These extracts were stored at −20 °C and used for the determination of the total phenolics, flavonoids, *ortho*-diphenols, and antioxidant activity (AA) assays.

#### 2.3.1. Total Phenolics

Total phenolic concentration was determined using the Folin–Ciocalteu colorimetric method with some modifications, described by Singleton and Rossi [23]. For that, 20 μL of extract was mixed with 100 μL of Folin–Ciocalteu reagent (1:10) and 80 μL of Na_2_CO_3_ (7.5%) in a 96-well microplate. The microplate was maintained in the dark for 30 min, and then, the absorbance values were obtained at 765 nm. Calibration was carried out using a gallic acid concentration curve, and the results were expressed as mg of gallic acid equivalents per g of dry weight (mg GAE g^−1^ of DW).

#### 2.3.2. Flavonoids

Flavonoid concentration was determined according to the colorimetric method described by Dewanto et al. [24] with some modifications. In a 96-well microplate, 100 μL of ddH_2_O, 10 μL of NaNO_2_ (5%), and 25 μL of extract were added. The plate was placed in the dark at room temperature for 5 min. Then, 15 µL of AlCl_3_ (10%) were added to each well. The plate was placed again in the dark for 6 min. Then, 50 μL of NaOH (1 M) and 50 μL of ddH_2_O were added, and the absorbance was read at 510 nm. A calibration curve was prepared with catechin, and the results were expressed as mg of catechin equivalents per g of dry weight (mg CE g^−1^ of DW).

#### 2.3.3. *Ortho*-Diphenols

The *ortho*-diphenols content was measured colorimetrically by reading the absorbance at 370 nm following the procedure described by Leal et al. [25] and Gouvinhas et al. [26]. For that, in a 96-well microplate, 160 μL of extract was mixed with 40 μL of sodium molybdate (5% *w*/*v*), and the plate was placed in the dark for 15 min. For calibration, a gallic acid curve was used, and the results were expressed as mg of gallic acid equivalents per g of dry weight (mg GAE g^−1^ of DW).

#### 2.3.4. Total Anthocyanins

The total monomeric anthocyanins (TMA) content was determined according to several authors [27,28,29]. To obtain the extracts, 50 mg of berries were added to 5 mL of methanol acidified with 1% HCl. The mixture was shaken and placed in the dark at 4 °C for 1 h. It was then centrifuged at 4000 rpm for 15 min at 4 °C, and the supernatant was collected. In a microplate, 50 μL of each extract was added to 250 μL of 0.025 M KCl (pH = 1.0) or 250 μL of 0.4 M sodium acetate buffer (pH = 4.5). Finally, absorbances of the mixtures with 0.025 M KCl and of the mixtures with 0.4 M sodium acetate buffer were read at 510 and 700 nm. The concentration of total monomeric anthocyanins was calculated according to the formula, TMA = (A*DF*MW)/(ɛ*C), where MW is the molecular weight of cyanidin-3-*O*-glucoside (449 g mol^−1^), DF is the dilution factor, ε is the molar extinction coefficient of cyanidin-3-*O*-glucoside (29,600), and C is the concentration of extracted volume and A = (A_510_ − A_700_)pH1.0 − (A_510_ − A_700_) pH4.5. Finally, results were expressed as mg of cyanidin-3-*O*-glucoside equivalents per gram of dry weight (mg CGE g^−1^ of DW).

### 2.4. Antioxidant Activity Assays

#### 2.4.1. ABTS^•+^ Radical-Scavenging Activity

To determine the radical-scavenging activity of berries extracts, the discoloration assay ABTS^•+^ (2,2′-azino-bis (3-ethylbenzothiazoline-6-sulphonic acid)) was used, as described by Re et al. and Stratil et al. [30,31]. For this, the ABTS^•+^ work solution was prepared using 7 mM ABTS mixed with 140 mM K_2_S_2_O_8_ in double distilled water. This mixture was then incubated for 12–16 h in the dark at room temperature, and its absorbance was adjusted with absolute ethanol to 0.7–0.8 at the wavelength of 734 nm. Following this, 15 µL of each berry extract (70% methanol (*v*/*v*) for the blank) plus 285 µL of the ABTS^•+^ work solution was mixed and left to stand for 10 min in the dark, after which absorbance was read at 734 nm. Results were expressed as µmol Trolox µg^−1^ of DW according to a Trolox calibration curve.

#### 2.4.2. DPPH Radical-Scavenging Activity

The reduction of the DPPH (2,2-diphenyl-1-picrylhydrazyl) radical was detected by measuring sample absorbance at 517 nm, according to several authors [32,33,34]. For this, 15 µL of extract (70% methanol (*v*/*v*) for the blank) were mixed with a 285 µL methanolic solution containing DPPH radicals (10^−5^ mol L^−1^). The mixture was vigorously shaken and left for 30 min in the dark. Using a Trolox calibration curve, the results were expressed as µmol Trolox µg^−1^ of DW.

#### 2.4.3. FRAP Assay

The FRAP (Ferric Reducing Antioxidant Power) assay used in this study was a modification of the previous method described by Stratil et al. and Benzie and Strain [31,35]. In sum, the FRAP reagent was prepared using 1 volume of an aqueous 10 mM solution of TPTZ (2,4,6-Tri(2-pyridyl)-s-triazine) in 40 mM HCl mixed with 1 volume of 20 mM FeCl_3_.6H_2_O and 10 volumes of 300 mM acetate buffer, pH 3.6. Then, 25 µL of berry extract (70% methanol (*v*/*v*) for the blank) were mixed with 275 µL of FRAP reagent. The mixture was vigorously shaken and left to stand for 5 min in the dark, followed by an absorbance reading at 593 nm. Using a Trolox calibration curve, the results were expressed as µmol Trolox µg^−1^ of DW.

### 2.5. Statistical Analysis

Data were analyzed using SPSS Statistics for Windows (IBM SPSS Statistics for Windows, Version 23.0, IBM Corp., Armonk, NY, USA). Statistical differences between treatments in each phenological stage of each year were evaluated by one-, two-, and three-way ANOVA, followed by Tukey multiple range test (*p* < 0.05). The results were presented as the mean (n = 30 for quality assessment of fruits or n = 3 for the determination of bioactive compounds) with the respective standard error (SE). A Pearson’s correlation analysis was used to determine the relationship between bioactive compound content and antioxidant activity values.

## 3. Results

### 3.1. Effect of Biostimulants on Berries Quality

To assess the influence of the seaweed extract (ANE) and glycine betaine (GB) in berry quality, several parameters were determined, namely fruit biometry (berry weight and dimensions), color, maturity index, and titratable acidity, at the veraison and harvest stages of the 2020 and 2021 growing seasons. In general, the biometric parameters were affected by the treatment (*p* < 0.001), year (*p* < 0.001), phenological stage (*p* < 0.001), the interaction between treatment and year (*p* < 0.05 for fruit height and *p* < 0.001 for the other biometric parameters), the interaction between treatment and phenological stage *p* < 0.05 for fruit height, *p* < 0.01 for weight and thickness, and *p* < 0.001 for width), and the interaction between year and phenological stage (*p* < 0.001), (Table A1 in Appendix A). Berries from grapevines sprayed with glycine betaine were heavier and bigger than those of the ANE treatments and C. In fact, for the veraison and harvest of 2020, treatments with GB 0.2% produced berries with improvements in the four biometric parameters analyzed (weight, height, width, and thickness) compared to the C berries (Table 1); for example, at the veraison of 2020, grapevines treated with GB 0.2% yielded berries with increased weight and dimensions, with these being, on average, 5% bigger than those of control plants.

At veraison of 2021, no significant differences were verified, except for height, where GB 0.2% presented an improvement of 1.8% in relation to C; the remaining treatments (ANE 0.05%, ANE 0.1%, and GB 0.1%) showed a slight decrease in this parameter. At the harvest of 2021, GB 0.1% showed improvements to the parameters weight (5.7%), width (4.1%), and thickness (3.1%), and GB 0.2% showed improvements to height (2.4%), compared to C.

**Figure 2:**
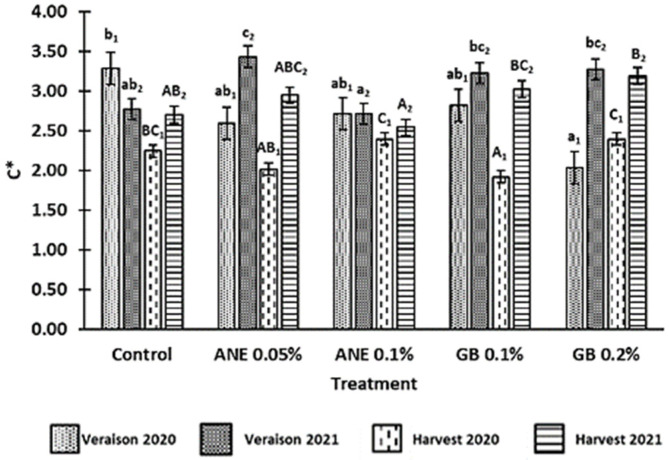
Chroma (C*) of berries of cv. ‘Touriga Franca’, with different treatments at veraison and harvests of 2020 and 2021. Values are means ± SE; different letters (lowercase—veraison; uppercase—harvest; 1—Year 2020; 2—Year 2021) mean significant differences between treatments within each phenological stage (*p* < 0.05, Tukey test). ANE—seaweed extract; GB—glycine betaine.

The values for the chroma (C*) of the berries in the two phenological stages and in both years are shown in Figure 2. It was verified that C* was affected by year (*p* < 0.001), phenological stage (*p* < 0.001), the interaction between treatment and year (*p* < 0.001), the interaction between treatment and phenological stage (*p* < 0.001), the interaction between year and phenological stage (*p* < 0.05), and the interaction between treatment, year, and phenological stage (*p* < 0.01) (Table A1 in Appendix A). In 2020, the lower C* value was observed in the berries treated with GB 0.2% at veraison and GB 0.1% at harvest. In 2021, berries from ANE 0.1% showed a lower C* value at veraison and harvest. At the harvest of both years, berries from GB 0.2% presented the highest C* value compared to C, with an increase of 15% in 2021.

**Figure 3:**
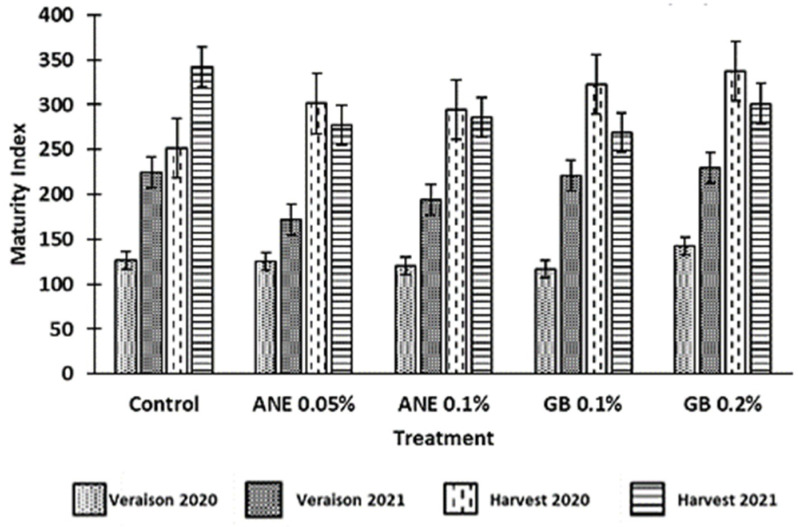
Maturity index (TSS*pH^2^) of berries of cv. ‘Touriga Franca’, with different treatments at veraison and harvests of 2020 and 2021. Values are means ± SE; no letters mean no significant differences between treatments within each phenological stage of each year (*p* < 0.05, Tukey test). ANE—seaweed extract; GB—glycine betaine.

The maturity index (MI) is calculated using the TSS (°Brix) and the pH values, being generally used to determine the optimum ripeness of red wine grapes. In this study, it verified an increase in MI from veraison to harvest (Figure 3), which is expected as the total soluble solids of berries tend to increase in this maturation period. However, no differences between treatments were verified at the statistical level (*p* > 0.05). Consequently, the application of ANE and GB did not affect the maturity index. However, berries from grapevines treated with GB 0.2% showed the highest values of MI in the 2020 harvest and in the veraison of both years, which could indicate that these grapevines were in a more advanced phenological stage (Figure 3).

**Figure 4:**
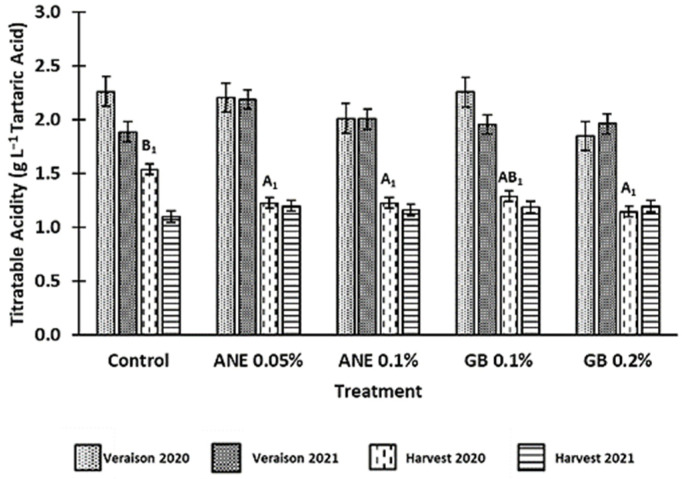
Titratable acidity (TA) of berries of cv. ‘Touriga Franca’, with different treatments at veraison and harvests of 2020 and 2021. Values are means ± SE; different letters (uppercase—harvest; 1—Year 2020) mean significant differences between treatments within each phenological stage of each year (*p* < 0.05, Tukey test), no letters mean no significant differences. ANE—seaweed extract; GB—glycine betaine.

The values for the titratable acidity (TA) of berries are shown in Figure 4. As expected, there was a decrease in TA from veraison to harvest in both years and in all treatments tested. Moreover, it was verified that TA was influenced by year (*p* < 0.05), phenological stage (*p* < 0.001), and the interaction between treatment and year (*p* < 0.05) (Table A1). In 2020, the values of TA were, on average, higher on both veraison (2.12 g·L^−1^ Tartaric Acid) and harvest (1.28 g·L^−1^ Tartaric Acid) compared to 2021, in which values were, on average, 2.00 g·L^−1^ Tartaric Acid at veraison and 1.17 g·L^−1^ Tartaric Acid at harvest.

Statistically significant differences between treatments (*p* < 0.05) were only observed for the harvest of 2020, where there was a reduction in berry TA for all treatments, but especially in berries of GB 0.2% (about 34% lower compared to the C).

### 3.2. Effects of Biostimulants on Berries Bioactive Compounds

**Figure 5:**
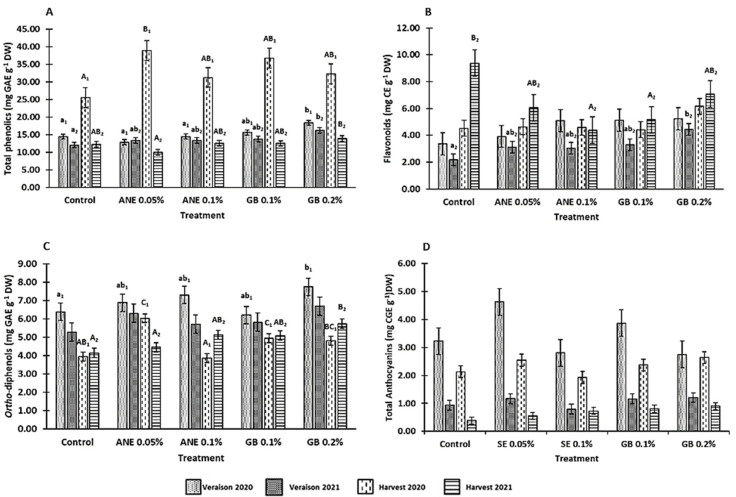
Variation on bioactive compound contents: total phenolics (**A**), flavonoids (**B**), *ortho*-diphenols (**C**), and total anthocyanins (**D**), in berries with different treatments in two consecutive years (2020 and 2021). Values are means ± SE; different letters (lowercase—veraison; uppercase—harvest; 1—Year 2020; 2—Year 2021) mean significant differences between treatments within each phenological stage of each year (*p* < 0.05, Tukey test), no letters mean no significant differences. ANE—seaweed extract; GB—glycine betaine.

The effect of seaweed extract (ANE 0.05% and ANE 0.1%) and glycine betaine (GB 0.1% and GB 0.2%) on bioactive compound contents was assessed via the determination of total phenolics, flavonoids, *ortho*-diphenols, and total anthocyanins (Figure 5).

It was verified that total phenolic content was affected by treatment (*p* < 0.001), year (*p* < 0.001), phenological stage (*p* < 0.001), the interaction between treatment and year (*p* < 0.05), the interaction between year and phenological stage (*p* < 0.001), and the interaction between treatment, year, and phenological stage (*p* < 0.05) (Table A1). Of all treatments, spraying with GB showed a higher improvement in this parameter (Figure 5A). At the veraison of 2020 and 2021, increases of 21% and 26% were observed in the berries treated with GB 0.2%, respectively. Also, at the 2021 harvest, berries sprayed with GB 0.2% showed the greatest increase in total phenolics, with the concentration being 12% higher than C. At the 2020 harvest, treatments with ANE 0.05% revealed higher increases in the concentration of total phenolics, 34% in comparison to the C, followed by a 31% increase with GB 0.1% and 21% in the spraying with GB 0.2%.

Looking at the concentration of flavonoids, this was not affected by year (*p* > 0.05), phenological stage (*p* > 0.05), the interaction between treatment and year (*p* > 0.05), the interaction between year and phenological stage (*p* > 0.05), nor the interaction between treatment, year, and phenological stage (*p* > 0.05), being only affected by treatment (*p* < 0.05) (Table A1). In the veraison and harvest of 2020, no significant differences (*p* > 0.05) were observed among treatments (Figure 5B). However, the treatment with GB 0.2% produced berries with slightly higher flavonoid content compared to the other treatments. In the growing season of 2021, opposite trends were observed at the veraison and harvest: at the 2021 veraison, both concentrations of GB and ANE increased the content of flavonoids in the berries compared to C (GB 0.2–51%; GB 0.1–33%; ANE 0.05–30% and ANE 0.1–28%); while for harvest, both biostimulants decreased the flavonoid concentration, with GB 0.2% (7.08 mg g^−1^) being the treatment with values closer to C (9.37 mg g^−1^).

The content of *ortho*-diphenols was affected by treatment (*p* < 0.001), year (*p* < 0.001), phenological stage (*p* < 0.001), the interaction between year and phenological stage (*p* < 0.001), and interaction between treatment, year, and phenological stage (*p* < 0.01) (Table A1). *Ortho*-diphenols content increased with GB 0.2% application at the veraisons of 2020 and 2021 (18% and 21% increase in relation to C, respectively) and at the harvest of 2021 (increase of 28% in relation to C) (Figure 5C). For the 2020 harvest, the concentration of *ortho*-diphenols increased with ANE 0.05% (35%), GB 0.1% (20%), and GB 0.2% (18%), in comparison to C.

The content of total anthocyanins (Figure 5D) was affected by the treatment (*p* < 0.05), year (*p* < 0.001), phenological stage (*p* < 0.001), and interaction between year and phenological stage (*p* < 0.01) (Table A1 in Appendix A). A high content of total anthocyanins was observed for the year 2020 compared to 2021 (3.3 and 3.7 times higher at veraison and harvest, respectively). The treatment of GB0.2%, in general (veraison of both years and harvest of 2021), increases the concentration of total anthocyanins compared to the control and other treatments.

### 3.3. Influence of Biostimulants on Antioxidant Potential

**Figure 6:**
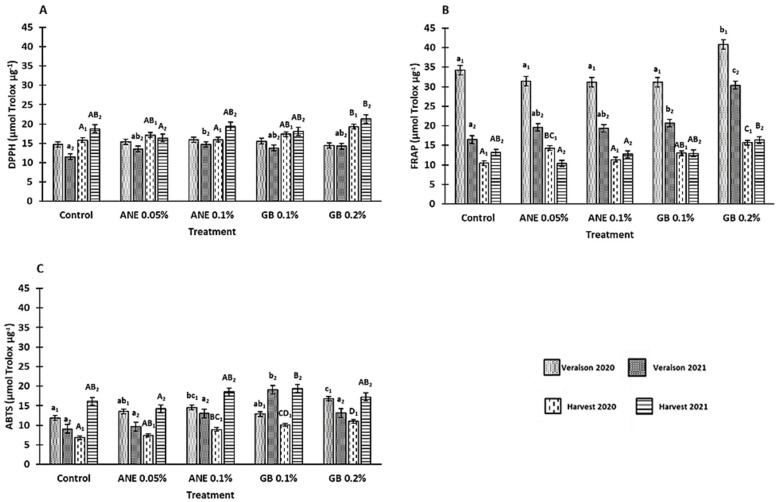
Antioxidant activity (AA): DPPH radical-scavenging activity (**A**), FRAP assay (**B**), and ABTS^•+^ radical-scavenging activity (**C**) in berries of cv. ‘Touriga Franca’, with different treatments in two consecutive years (2020 and 2021). Values are means ± SE; different letters (lowercase—veraison; uppercase—harvest; 1—Year 2020; 2—Year 2021) mean significant differences between treatments within each phenological stage of each year (*p* < 0.05, Tukey test), no letters mean no significant differences. ANE—seaweed extract; GB—glycine betaine.

In the methods used to verify the influence of biostimulant treatments in the antioxidant activity (AA) of the berries, the DPPH method was influenced by the treatment (*p* < 0.001), phenological stage (*p* < 0.001), interaction between treatment and phenological stage (*p* < 0.05), and interaction between year and phenological stage (*p* < 0.001) (Table A1). The FRAP and ABTS^•+^ methods showed differences between the treatment (*p* < 0.001), years (*p* < 0.001), phenological stage (*p* < 0.01 for ABTS^•+^ and *p* < 0.001 for FRAP), and interaction between year and phenological stage (*p* < 0.001) (Table A1). Moreover, significant differences (*p*< 0.05) for the berry’s AA (by FRAP, ABTS^•+^, and DPPH methods) were found between treatments at the veraison and harvest of both years (except in DPPH at veraison 2020). In general, berries from grapevines treated with GB presented the highest AA (Figure 6). At veraison 2020, berries of GB 0.2% showed an increase in AA (16% for FRAP and 29% for ABTS^•+^ methods) (Figure 6B,C). Furthermore, at the veraison of 2021, berries of GB 0.2% showed a 46% increase in the analysis via the FRAP method, and berries of GB 0.1% revealed an increase of 52% via the ABTS^•+^ method. For the harvests of 2020 and 2021, the treatment with GB 0.2% increased the AA (by DPPH and FRAP methods) in comparison to C (18% and 12% for DPPH and 33% and 19% for FRAP, respectively (Figure 6A,B). The analysis of AA via the ABTS^•+^ method on GB 0.2% treated berries also revealed an increase of 38% for the harvest of 2020 and an increase of 17% on GB 0.1% treatment at the harvest of 2021 (Figure 6C).

## 4. Discussion

### 4.1. Application of Biostimulants Positively Affected Berry Quality

The foliar application of biostimulants, namely *A. nodosum* extracts and glycine betaine, could be a good strategy to improve grapevine’s resilience to climate change in many wine regions around the world, especially because these products are low cost and eco-friendly. Several studies with different species have shown that the applications of ANE and GB can increase the physical and chemical attributes of fruits [8,9,10,11,12,16,36,37,38]. Some studies in grapevine report that the application of ANE leads to anthocyanin accumulation while also increasing phenolic, flavonols, and tannins contents [8,11,12,16]. Furthermore, the application of GB in strawberries increased plant growth and yield under deficit irrigation conditions; in the case of cv. Fortuna, it also improved fruit firmness, chroma, and total anthocyanins; in cv. Albion, it increased total soluble solids and ascorbic acid content [9]. In sweet cherry, the application of GB with calcium improved visual appearance, and color [10]. In other studies with GB, it was revealed it enhanced the growth and productivity of cucumber under drought [37], while in olive, it was observed to increase production values [38].

Berries of grapevines sprayed with GB were bigger and heavier than those of the treatments with ANE and C (Table 1). Similar results were found in the sweet cherry cvs. ‘Skeena’ and ‘Sweetheart’, where GB sprayings increased fruit weight [10] and increased fruit dimensions in the cv. ‘Staccato’ [39]. Olive caliber has also been observed to improve with the application of GB [38]. Comparatively, GB applications in sunflowers also revealed favorable effects on the weight of the achenes [36]. Adak [9] also observed that strawberry plants treated with GB had increased the crown diameter and fruit weight.

Color is generally considered one of the bases for quality assessment, not only due to its aesthetic role and nutritional value but also due to the influence that grape pigments have on the wine color [40]. The parameter C* refers to color saturation; with lower C* values being associated with colored berries, while higher C* values are linked to non-colored ones [9,40]. In a previous study by Correia et al. [10], the authors observed a decrease in the C* of cherries treated with GB 0.1% in comparison to the control, which was also verified in this study for the grape harvest of 2020, using the same spraying concentration (Figure 2). Despite this, GB 0.2% led to an increase in C* compared to the control at the harvests of both years. Similar results were previously found in strawberries (cv. Fortuna) using different concentrations of GB [9]. Nonetheless, this increase in C* could also be associated with the fact that these berries treated with GB0.2% presented a higher maturation index (MI) (Figure 3). The optimal values of MI range from 200 to 270 at harvest [22]. However, in this present study, the MI values at harvest were above this range, averaging 300 in 2020 and 295 in 2021 (Figure 3). The MI is significantly influenced by the weather conditions of the growth year, as verified by Rätsep et al. [41] in grapevine cv. Zilga. In fact, we verified that, in this work, the MI was affected by the year (*p* < 0.01), the phenological stage (*p* < 0.001), and the interaction between the year and phenological stage (*p* < 0.001) (Table A1). In the *Douro Superior* region, the year of 2020 was considered hot and dry. In particular, the month of July was extremely hot and dry (Figure 1), being regarded as the hottest since 1931, according to IPMA (*Instituto Português do Mar e da Atmosfera*), which contributed to the occurrence of grapevine sunburn [42]. On the other hand, 2021 was perceived as a normal and dry year [43] (Figure 1). This phenomenon may explain why the berries of 2020 berries had a higher MI. Although there were no significant differences at the statistical level, we observed an increase in MI at the harvest of 2020 and veraison of both years when GB 0.2% was applied, in comparison to control treatment (Figure 3). Similar effects of GB spraying in the MI have been previously observed by Metwaly et al. [37] in cucumber.

It is known that acidity is influenced by radiation, temperature, and water availability [44]. This might explain why we observed lower acidity values in 2021 (Figure 4), considering it was a year with higher temperatures and lower precipitation levels (Figure 1).

### 4.2. Application of Biostimulants Positively Affected Berry Bioactive Compounds and Antioxidant Activity

It is known that climate conditions are the driving factor influencing grape and wine quality [45,46]. Temperatures are rising worldwide, and most regions are being increasingly exposed to prolonged water deficit periods [46]. In fact, during the veraison of 2020, the precipitation levels were lower than in the veraison of 2021 (Figure 1). The average temperature in July 2020 was 28.8 °C [42], while in 2021, it was 24.7 °C [43]. The high temperatures, along with the low precipitation values, influenced the synthesis of bioactive compounds [44,46], leading to the increase verified in the veraison of 2020. This is quite noticeable in the total anthocyanins content in the veraison of 2020, which was 3.3 times higher than in 2021 (Figure 5D). Regarding the total phenolics, flavonoids, and *ortho*-diphenols in the veraison of 2020, the contents were, on average, 1.2 times higher than in the veraison of 2021 (Figure 5). Moreover, a higher total phenolics content was observed in berries sprayed with GB compared with the other treatments and control. These results were consistent with previous studies, namely those of Awad et al. [47], with postharvest application of GB in table grapes cv. El-Bayadi; Khadouri et al. [14], with cowpea under water stress; Shafiq et al. [48], with maize under water stress; and Safwat et al. [49], in basil under salt stress. An opposite effect was verified for ANE 0.05% at the veraison of 2020 and the harvest of 2021, where a lower total phenolic content was observed. A similar decrease in total phenolics was observed in cv. Merlot after the foliar application of *A. nodosum* extract at the lowest tested concentration [12]. At the harvest of 2020, ANE seemed to improve the total phenolics content, being in agreement with the studies of Frioni et al. [8] in cv. Sangiovese, Cabo et al. [13] in hazelnut and Rouphael et al. [50] in spinach leaves. In this study, an increase in the *ortho*-diphenols content was observed in the berries of grapevines with the foliar spraying of GB and ANE. Similarly, Cabo et al. [13] also verified an increase in the concentration of *ortho*-diphenols in hazelnuts after the foliar application of ANE. A similar trend for the concentration of total phenols and *ortho*-diphenols was observed at the harvest of 2020, where the treatments with ANE 0.05%, GB 0.1%, and GB 0.2% revealed increases in both concentrations in comparison to the C.

In the case of flavonoids, both biostimulants appeared to increase their concentration at the veraison of 2021, which is in line with other studies, namely in the postharvest treatment of table grapes cv. El-Bayadi with GB [47], in sweet cherry with foliar application of GB [7], in cv. Sangiovese was sprayed with ANE [11], and hazelnuts were sprayed with ANE [51].

In general, the foliar application of GB in grapevine tends to increase the concentration of bioactive compounds (total phenolics, flavonoids, and *ortho*-diphenols), mainly at the veraison stage, with the highest concentration (GB 0.2%) being the most promising for the grape cultivar ‘Touriga Franca’.

Like the results obtained for the bioactive compounds, antioxidant activity was also observed to be increased in the berries of grapevines subjected to GB foliar applications (Figure 6). In fact, a positive correlation between bioactive compounds and antioxidant activity was observed in this study, with total phenolics being the parameter with a better correlation with AA (Table A2 in Appendix B). Indeed, at the veraison of 2020, DPPH values were positively correlated with total phenolics (R^2^ = 0.630; *p* < 0.01) and flavonoids (R^2^ = 0.334; *p* < 0.05); FRAP values were positively correlated with total phenolics (R^2^ = 0.646; *p* < 0.01), flavonoids (R^2^ = 0.374; *p* < 0.05), and *ortho*-diphenols (R^2^ = 0.650; *p* < 0.01); and ABTS^•+^ values were positively correlated with total anthocyanins (R^2^ = 0.395; *p* < 0.01). At the veraison of 2021, positive correlations were also observed between DPPH values and total phenolics (R^2^ = 0.575; *p* < 0.01); and between FRAP values and total phenolics (R^2^ = 0.749; *p* < 0.01) and *ortho*-diphenols (R^2^ = 0.475; *p* < 0.01). At the harvest of 2020, DPPH and FRAP values had significant (*p* < 0.01) positive correlations with *ortho*-diphenols and total anthocyanins. At the harvest of 2021, FRAP values showed a positive correlation (*p* < 0.01) with total phenolics and *ortho*-diphenols, while DPPH and ABTS^•+^ values showed a statistically significant (*p* < 0.05) positive correlation with *ortho*-diphenols and total phenolics, respectively.

## 5. Conclusions

In this study, the differences detected between 2020 and 2021 in the berry quality parameters may reflect the variation observed in the climatic conditions between the years, further corroborating that the climate plays a key role when growing high-quality grapes. The foliar application of *A. nodosum* extract (ANE) and glycine betaine (GB) improved the physiological and biochemical performance of grapevine cv. ‘Touriga Franca’ exposed to the summer stress in the Douro Demarcated Region, sub-Region ‘*Douro Superior*’; sprayings with these biostimulants have shown to be a promising strategy in the mitigation of the effects of summer stress in the grapevine cv. ‘Touriga Franca’ in this region, with special emphasis on GB, led to higher contents of bioactive compounds (total phenolics, flavonoids, and *ortho*-diphenols). Therefore, these biostimulants could be an affordable climate change mitigation tool for viticulturists worldwide while also improving berry quality. However, further studies are still needed in order to confirm the effects observed in this study in different viticultural regions and/or different varieties.

## Figures and Tables

**Table 1:** Biometric parameters: weight, height, width, and thickness of 30 berries of cv. ‘Touriga Franca’, with different treatments, at veraison and harvest of 2020 and 2021. Values are means ± SE, different letters (lowercase—veraison; uppercase—harvest; 1—Year 2020; 2—Year 2021) mean significant differences between treatments within each phenological stage of each year (*p* < 0.05, Tukey test). C—control; ANE—seaweed extract; GB—glycine betaine.

Biometric Parameters	Growth Stage/Year	C	ANE 0.05%	ANE 0.1%	GB 0.1%	GB 0.2%
Weight (g)	Veraison 2020	1.92 ± 0.05 b_1_	1.90 ± 0.05 b_1_	1.86 ± 0.05 ab_1_	1.71 ± 0.04 a_1_	2.02 ± 0.04 b_1_
	Veraison 2021	2.11 ± 0.04	1.99 ± 0.05	2.10 ± 0.04	2.13 ± 0.04	2.14 ± 0.04
	Harvest 2020	2.09 ± 0.04 A_1_	2.09 ± 0.04 A_1_	2.04 ± 0.05 A_1_	2.09 ± 0.05 A_1_	2.28 ± 0.05 B_1_
	Harvest 2021	2.14 ± 0.04 BC_2_	1.83 ± 0.06 A_2_	2.08 ± 0.05 B_2_	2.27 ± 0.04 C_2_	2.23 ± 0.04 BC_2_
Height (mm)	Veraison 2020	14.15 ± 0.14 a_1_	14.21 ± 0.13 a_1_	14.63 ± 0.17 ab_1_	14.18 ± 0.12 a_1_	15.07 ± 0.11 b_1_
	Veraison 2021	15.41 ± 0.10 c_2_	14.78 ± 0.13 a_2_	15.36 ± 0.15 bc_2_	14.94 ± 0.08 ab_2_	15.70 ± 0.14 c_2_
	Harvest 2020	14.83 ± 0.13 A_1_	14.71 ± 0.13 A_1_	15.00 ± 0.16 AB_1_	15.03 ± 0.15 AB_1_	15.39 ± 0.12 B_1_
	Harvest 2021	16.51 ± 0.13 B_2_	15.47 ± 0.20 A_2_	16.44 ± 0.17 B_2_	16.62 ± 0.14 B_2_	16.91 ± 0.12 B_2_
Width (mm)	Veraison 2020	13.91 ± 0.12 a_1_	14.06 ± 0.12 ab_1_	14.11 ± 0.13 ab_1_	13.72 ± 0.12 a_1_	14.43 ± 0.10 b_1_
	Veraison 2021	14.73 ± 0.10	14.58 ± 0.12	14.88 ± 0.11	14.90 ± 0.10	14.83 ± 0.11
	Harvest 2020	14.20 ± 0.11 AB_1_	14.12 ± 0.11 AB_1_	13.81 ± 0.14 A_1_	13.86 ± 0.12 A_1_	14.33 ± 0.12 B_1_
	Harvest 2021	14.13 ± 0.12 B_2_	13.29 ± 0.18 A_2_	13.87 ± 0.13 B_2_	14.73 ± 0.12 C_2_	14.36 ± 0.11 BC_2_
Thickness (mm)	Veraison 2020	13.45 ± 0.12 a_1_	13.58 ± 0.12 ab_1_	13.61 ± 0.13 ab_1_	13.25 ± 0.12 a_1_	13.99 ± 0.10 b_1_
	Veraison 2021	14.20 ± 0.09	14.22 ± 0.12	14.47 ± 0.11	14.43 ± 0.10	14.32 ± 0.12
	Harvest 2020	13.71 ± 0.10 AB_1_	13.59 ± 0.10 AB_1_	13.28 ± 0.14 A_1_	13.45 ± 0.13 AB_1_	13.84 ± 0.13 B_1_
	Harvest 2021	13.49 ± 0.14 BC_2_	12.82 ± 0.17 A_2_	13.26 ± 0.14 AB_2_	13.92 ± 0.11 C_2_	13.60 ± 0.12 BC_2_

## Data Availability

Data is contained within the article.

## Appendix A

**Table A1:** Interactions at statistical level of treatment (T), year (Y), and phenological stage (PS) of each parameter analyzed.

Parameter	*P* (T)	*P* (Y)	*P* (PS)	*P* (T*Y)	*P* (T*PS)	*P* (Y*PS)	*P* (T*Y*PS)
Weight	<0.001	<0.001	<0.001	<0.001	<0.01	<0.001	>0.05
Height	<0.001	<0.001	<0.001	<0.05	<0.05	<0.001	>0.05
Width	<0.001	<0.001	<0.001	<0.001	<0.001	<0.001	<0.05
Thickness	<0.001	<0.001	<0.001	<0.001	<0.01	<0.001	>0.05
Chroma	>0.05	<0.001	<0.001	<0.001	<0.001	<0.05	<0.01
Maturity index	>0.05	<0.01	<0.001	>0.05	>0.05	<0.001	>0.05
Titratable acidity	>0.05	<0.05	<0.001	>0.05	>0.05	>0.05	>0.05
Total Phenolics	<0.001	<0.001	<0.001	<0.05	>0.05	<0.001	<0.05
Flavonoids	<0.05	>0.05	>0.05	>0.05	>0.05	>0.05	>0.05
*Ortho*-diphenols	<0.001	<0.001	<0.001	>0.05	>0.05	<0.001	<0.01
Total Anthocyanins	<0.05	<0.001	<0.001	>0.05	>0.05	<0.01	>0.05
DPPH	<0.001	>0.05	<0.001	>0.05	<0.05	<0.001	>0.05
FRAP	<0.001	<0.001	<0.001	>0.05	<0.001	<0.001	>0.05
ABTS^•+^	<0.001	<0.001	<0.01	>0.05	>0.05	<0.001	>0.05

## Appendix B

**Table A2:** Correlation matrix (R^2^ and *P* values) established between bioactive compounds and antioxidant activity in berries of cv. ‘Touriga Franca’ at veraison and harvests of 2020 and 2021. * Correlation is significant at *p* < 0.05; ** correlation is significant at *p* < 0.01.

			Total Phenolics	Flavonoids	*Ortho*-Diphenols	Total Anthocyanins
Veraison 2020	DPPH	R^2^	0.630 **	0.334 *	0.221	0.113
		*P*	0.000	0.025	0.145	0.461
	FRAP	R^2^	0.646 **	0.374 *	0.650 **	−0.111
		*P*	0.000	0.011	0.000	0.467
	ABTS•+	R^2^	0.198	0.166	−0.083	0.395 **
		*P*	0.192	0.276	0.588	0.007
Veraison 2021	DPPH	R^2^	0.575 **	−0.079	0.215	0.094
		*P*	0.000	0.608	0.156	0.538
	FRAP	R^2^	0.749 **	−0.004	0.475 **	0.152
		*P*	0.000	0.977	0.001	0.318
	ABTS•+	R^2^	0.045	−0.030	−0.218	0.076
		*P*	0.771	0.843	0.150	0.619
Harvest 2020	DPPH	R^2^	0.063	0.109	0.414 **	0.412 **
		*P*	0.679	0.476	0.005	0.005
	FRAP	R^2^	0.134	0.047	0.488 **	0.467 **
		*P*	0.381	0.757	0.001	0.001
	DPPH	R^2^	0.070	−0.021	0.020	−0.001
		*P*	0.647	0.890	0.897	0.997
Harvest 2021	FRAP	R^2^	0.525 **	0.023	0.332 *	−0.038
		*P*	0.000	0.880	0.026	0.806
	ABTS•+	R^2^	0.785 **	0.017	0.677 **	−0.132
		*P*	0.000	0.912	0.000	0.387
	FRAP	R^2^	0.321 *	0.085	0.151	0.162
		*P*	0.032	0.578	0.322	0.288
